# Supercritical Carbon Dioxide Extraction of *Nicotiana tabacum* Leaves: Optimization of Extraction Yield and Nicotine Content

**DOI:** 10.3390/molecules27238328

**Published:** 2022-11-29

**Authors:** Nina Djapic

**Affiliations:** Technical Faculty “Mihajlo Pupin”, University of Novi Sad, Djure Djakovica bb, 23000 Zrenjanin, Serbia; nina.djapic@tfzr.rs

**Keywords:** *Nicotiana tabacum*, nicotine, supercritical CO_2_ extraction

## Abstract

**Highlights:**

Nicotine was extracted from varieties Samsun and Virginia tobacco leaves using supercritical CO_2_ extraction.
Response surface methodology was used to find factors that influence the supercritical CO_2_ extraction of tobacco leaves.
The highest nicotine relative amount was at lower pressure (15 MPa), temperature of 50 °C and during 90 min extraction time.
The quantity of nicotine and phytochemical composition in different tobacco varieties is specific.

**Abstract:**

The employment of supercritical carbon dioxide extraction for obtaining the chemical compounds from *N. tabacum* leaves, especially nicotine, is advancing. The supercritical carbon dioxide extraction of dried *N. tabacum* cv. Samsun and *N. tabacum* cv. Virginia at different process parameters was performed to obtain the highest extraction yield and nicotine relative amount. The optimal extraction conditions concerning the highest extraction yield and nicotine relative amount were determined by response surface methodology. The highest extraction yield for *N. tabacum* cv. Samsun was 2.99% and for *N. tabacum* cv. Virginia 2.33% at 23.41 MPa, 50 °C and 90 min of extraction time. The highest nicotine relative amount in *N. tabacum* cv. Samsun and *N. tabacum* cv. Virginia was at 15 MPa, 50 °C and 90 min extraction time and was 242.1 mg per 100 g of plant material and 32.4 mg per 100 g of plant material, respectively. The pressure, temperature and time influenced the extraction yield and nicotine relative amount recovery in *N. tabacum* cv. Samsun and *N. tabacum* cv. Virginia. A general inclusive concept in respect to pressure, temperature and time of the supercritical carbon dioxide extraction and a report on phytochemicals present in two *N. tabacum* varieties is presented.

## 1. Introduction

*Nicotiana tabacum* L., Solanaceae is the most common tobacco with approximately 152 cultivated varieties [1]. The main cultivated tobacco species are Burley, Virginia, and Oriental [2,3]. The Oriental tobacco, which includes several hundred varieties, has four primary groups: Samsun, Smyrna, Kavalla, and Xanthi [2]. The chemical constituents of tobacco leaf and differences among tobacco types are well described [4]. *N. tabacum* leaves are the source of nicotine [4,5]. Nicotine can range in concentrations from 0.5 to 8% in cultivated tobacco species [4]. Nicotine is soluble in alcohol, chloroform, ether, petroleum ether, kerosene, and water [6,7]. Various solvents can be used to isolate nicotine from tobacco leaves using the solvent extraction method [6,7]. The separation of nicotine from tobacco leaves can be performed with supercritical carbon dioxide (SC CO_2_) [8]. In the single-stage process at the pressure of approximately 30 MPa and the temperatures between the critical temperature of the gas and 100 °C, the dissolved nicotine is separated by reducing the temperature or by changing the temperature or is bound by adsorption onto suitable sorbents [8]. The residual nicotine content as a function of processing time for Burley, Virginia and Oriental tobacco leaves is different [8]. Tobaccos of different origin behave differently under the SC CO_2_ extraction [9,10]. The supercritical fluid extraction was used for the extraction of *N. tabacum* leaves and apart from nicotine and solanesol, in those extracts, α-tocopherol was detected [11,12]. The SC CO_2_ is used in the tobacco processing industry for removing nicotine and producing low-nicotine tobacco. The reduction in nicotine content in tobacco is completed in several stages [10]. After the selective removal of aroma with the SC CO_2_, the obtained aroma is used to impregnate a previous batch from which the nicotine and aroma have been removed [10]. This is completed by allowing the SC phase to expand into the batch [10]. The de-aromatized tobacco is moistened, and the nicotine is removed in an isobaric and isothermal recycling operation involving a selective sorbent, indicating that the moisture is the essential in the extraction of nicotine [10]. After all the stages, the nicotine content of the tobacco is reduced to ~95% [10]. The mixture of tobacco varieties, in form of 32.5% flue-cured, 19.9% Burley, 1.2% Maryland, 11.1% Oriental and 27.1% reconstituted, has been used for the SC CO_2_ extraction of nicotine under the pressure of 26 MPa and temperature of 70 °C [10]. The influence of particle size, cell geometry and packing of the extraction cell was investigated for the extraction of nicotine from the tobacco using cosolvent, 12 cm^3^ MeOH:41.2 mM KH_2_PO_4_ = 2:3, under the pressure of 13.7 MPa, temperature of 100 °C for 35 min [13]. Tobacco waste, derived from tobacco leaves and obtained during tobacco processing, has been used for the extraction of nicotine by SC CO_2_ extraction [14]. The SC CO_2_ extraction conditions were at the pressure from 15 to 30 MPa, time was from 180 to 300 min and temperature was from 50 to 70 °C [14].

Many factors influence the SC CO_2_ extraction, and it is important to screen the factors that influence the SC CO_2_ process to find the responses. The response surface methodology (RSM) and central composite rotatable design (CCRD) are appropriate to find the important factors influencing the process. This optimization design permits finding the optimal levels of chosen factors that influence the process in SC CO_2_ [15]. The RSM is useful for modeling and analysis of factors where a response of interest is influenced by several variables [16,17]. The present research aims to optimize the pressure, temperature and time for the production of high extraction yield and nicotine content of two *N. tabacum* varieties. From the Oriental tobacco leaves, chosen was the variety Samsun grown in Shuakhevi, Adjara, Georgia, and the other variety was Virginia, which is the most commonly grown of all plants in the genus *Nicotiana*; the leaves grown to be processed into tobacco were grown in Kukujevci, Srem, Serbia. 

Starting from the assumption that Oriental and flue-cured tobacco leaves have different aroma, chosen were *N. tabacum* cv. Samsun and *N. tabacum* cv. Virginia leaves for this research. The aim of the work was to investigate the (1) influence of process parameters, pressure, temperature, and time on SC CO_2_ extraction of dried *N. tabacum* cv. Samsun and *N. tabacum* cv. Virginia on the total extraction yield; (2) chemical profile of extracts analyzed by GC–MS; and (3) influence of process parameters, pressure, temperature, and time on SC CO_2_ extraction of dried *N. tabacum* cv. Samsun and *N. tabacum* cv. Virginia on the relative amount of nicotine analyzed by GC–MS. In addition, the aim was also to (4) determine optimal extraction conditions by RSM.

## 2. Results

### Optimization of SC CO_2_ Extraction of N. tabacum cv. Samsun and N. tabacum cv. Virginia Leaves

The CCRD was used to optimize the operating variables, pressure, temperature, and time of the SC CO_2_ extraction to achieve the highest extraction yield and higher relative amount of nicotine from the *N. tabacum* cv. Samsun and *N. tabacum* cv. Virginia leaves. The design matrix indicating the coded variables is depicted in Table 1.

The CCRD was completed with 20 experiments where six replicates were for the central point (Table 2).

The effects of linear, square, and two-way interaction coefficients on the response were tested for the significance by the analysis of variance (ANOVA). Regression coefficients of constant, linear, square and interaction terms of the model were obtained using the least square method. The degree of significance is determined by the *p*-value (Table 3).

The ANOVA results for the extraction yield for the *N. tabacum* cv. Samsun and *N. tabacum* cv. Virginia leaves total extraction yield [%] are depicted in Table 4.

**Figure 1:**
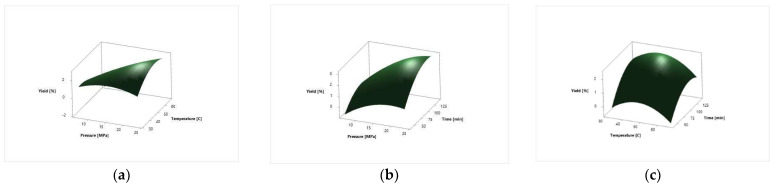
Surface response plots for the *N. tabacum* cv. Samsun leaves extraction yield in a function of extraction: (**a**) pressure and temperature; (**b**) pressure and time and (**c**) temperature and time.

The surface response plots for the effect of independent variables on the *N. tabacum* cv. Samsun leaves total extraction yield [%] are depicted in Figure 1.

**Figure 2:**
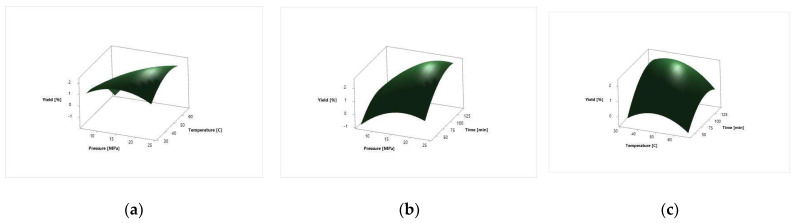
Surface response plots for the *N. tabacum* cv. Virginia leaves extraction yield as a function of extraction; (**a**) pressure and temperature; (**b**) pressure and time; and (**c**) temperature and time.

The surface response plots for the effect of independent variables on the *N. tabacum* cv. Virginia leaves total extraction yield [%] are depicted in Figure 2.

The CCRD was used to optimize the extraction pressure, temperature and time to achieve the highest relative amount of nicotine. The chemical profiles of *N. tabacum* cv. Samsun and *N. tabacum* cv. Virginia leaves’ extracts analyzed by GC–MS and their relative amount are depicted in Table 5 and Table 6, respectively.

The ANOVA was used for the calculation of regression coefficients of constant, linear, square and interaction terms of the nicotine relative amount model for *N. tabacum* cv. Samsun and *N. tabacum* cv. Virginia leaves’ extracts (Table 7).

The ANOVA results for the nicotine relative amount for the *N. tabacum* cv. Samsun and *N. tabacum* cv. Virginia leaves are depicted in Table 8.

**Figure 3:**
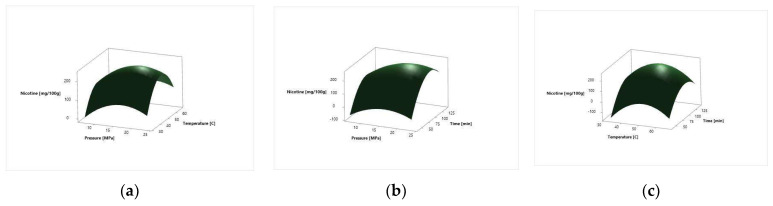
Surface response plots for the *N. tabacum* cv. Samsun leaves nicotine relative amount in a function of extraction: (**a**) pressure and temperature; (**b**) pressure and time; and (**c**) temperature and time.

**Figure 4:**
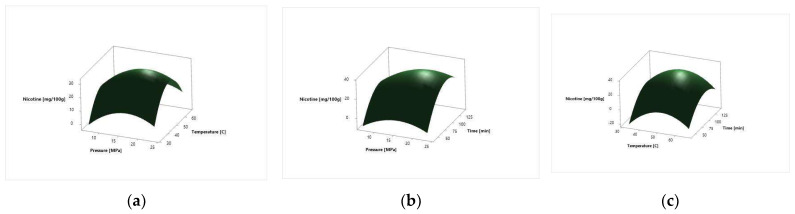
Surface response plots for the *N. tabacum* cv. Virginia leaves nicotine relative amount in a function of extraction: (**a**) pressure and temperature; (**b**) pressure and time; and (**c**) temperature and time.

The surface response plots, for the effect of independent variables on the *N. tabacum* cv. Samsun and *N. tabacum* cv. Virginia leaves total nicotine relative amount, are depicted in Figure 3 and Figure 4, respectively.

## 3. Discussion

The extraction yield of *N. tabacum* cv. Samsun leaves varied from 0.11% to 2.99% under applied process parameters (Table 2). The lowest yield was at the pressure of 10 MPa, temperature of 60 °C and 60 min extraction time. The highest yield was at the pressure of 23.41 MPa, temperature of 50 °C and 90 min extraction time. The extraction yield of *N. tabacum* cv. Virginia was from 0.08% to 2.33% (Table 2). The extraction yield, for both plant systems analyzed, increased with the increase in pressure. The linear term of pressure, temperature and time, the square term of pressure, temperature and time, and two-way interaction factor of pressure and temperature exhibited the most statistically significant influence (*p* < 0.05) on the extraction yield in both plant systems (Table 3). The two-way interaction factors of pressure and time and temperature and time did not have a significant influence on the extraction process for the total extraction yield in both plant systems analyzed. The visual effects of independent on dependent variables are depicted on the surface response plots of the proposed model in Figure 1 and Figure 2. From the surface response plots, it can be seen that the extraction yield increases with the extraction time. The similar shape of response plots 1a and 2a, 1b and 2b, and 1c and 2c indicate that the influence of process parameters is almost the same on the total extraction yield in both plant varieties analyzed.

The obtained SC CO_2_ extracts were characterized by gas chromatography-mass spectrometry (GC–MS). The main compound in Samsun tobacco leaves was nicotine, and different extraction parameters influenced its relative amount in the extracts (Table 5). Nicotine is the major alkaloid in tobacco leaves [4]. Its quantity depends on the variety, climate conditions, cultivation, and processing methods [4]. Neophytadiene is present in Samsun tobacco leaves in quantities from 17.25 to 38.29 mg expressed as nicotine equivalents per 100g of plant material. Its quantities are the highest in Burley tobacco leaves [18]. The main compound in Virginia tobacco leaves was 8,13-epoxy-14-labden-12-ol, a tricyclic diterpenoid, also identified in *N. tabacum* Oriental type tobacco leaves Yaka and Prilep, and semi Oriental type Otlja [19]. The main compounds in Virginia tobacco leaves, apart from a tricyclic diterpenoid were nicotine, a primary alcohol solanesol, neophytadiene, a sesquiterpenoid farnesol, unsaturated ketone solanone and a diketone norsolanadione (Table 6). Other compounds identified in Virginia tobacco leaves were present only under some extraction parameters (Table 6). The compounds detected in both plant varieties leaves were nicotine, neophytadiene, sesquiterpenoid 3-oxo-α-ionol, a monocyclic diterpene alcohol thunbergol, and sesquiterpene lactone sclareolide (Table 6).

Different SC CO_2_ extraction parameters influence the abundance of compounds present in the SC CO_2_ extracts. The effects of linear, square, and two-way interaction on the nicotine relative amount in Samsun and Virginia tobacco leaves are depicted in Table 7 and Table 8, respectively. The regression coefficients of intercept, linear, square, and two-way interaction terms of the model were determined by the least square method. The degree of significance of every factor is represented with *p*-factor. For both systems analyzed, the linear term of pressure and time and all square terms showed the most significant influence. The linear term of temperature and the two-way interactions did not exhibit a statistically significant influence on any of the investigated responses (Table 7). The coefficient of determination *R*^2^ was 0.9659 for Samsun and 0.9514 for Virginia, indicating that the model was made with satisfactory coefficients of determination. The data obtained were used to create the three-dimensional graphs of the response surface (Figure 3 and Figure 4). The similar shape of the response plots 3a and 4a, 3b and 4b, and 3c and 4c indicated the same influence of process parameters on the extraction of nicotine relative amount. The SC CO_2_ extraction has been used for removing nicotine [20]. It was proposed that the moisture and compounds present in plant material influence the extraction yield of nicotine [13]. The results obtained indicated that the pressure has a significant influence on nicotine yield [11,14,21]. The extraction yield of nicotine from tobacco waste, which also contains leaves’ particles, increases with the increase in pressure and at the temperatures between 50 and 60 °C [14]. At temperatures above 60 °C, the extraction yield of nicotine decreases [14]. The optimal temperature for Samsun and Virginia leaves was 50.51 °C, indicating the accordance with previous investigations [14]. The extraction at higher temperatures yields extracts with high nicotine content, while extractions at lower temperatures yield extracts with high solanesol content [11]. One investigation suggested that for the extraction of nicotine from tobacco leaves, higher pressures are favorable due to the selectivity and high extraction yield [14]. For Samsun leaves, the optimal pressure was 17.80 MPa and for Virginia, it was 17.29 MPa for obtaining the high nicotine relative amount. In previous investigations, the highest nicotine relative content in *N. tabacum* leaves was obtained at pressure of 15 MPa and at the temperature of 50 °C [11]. The lowest nicotine relative content was, in one investigation, at 8 MPa and temperature of 25 °C, indicating that the lower temperatures influenced the relative nicotine content in the extract [11]. The study on nicotine content in *N. tabacum* L. leaves was in one investigation 19.34% (15 MPa and 28 °C) and 23.70% (15 MPa and 60 °C); 12.29% (17 MPa and 60 °C) and 22.50% (17 MPa and 80 °C); and 47.40% (12 MPa and 60 °C) and 25.87% (12 MPa and 80 °C) [12]. The highest extraction yield was at lower pressure, the pressure of 12 MPa and temperature of 60 °C [12]. The lowest nicotine content was obtained at 17 MPa and at the temperature of 60 °C, indicating that the increase in pressure at the same temperature decreases the nicotine content [12]. This suggested explanation that with the increase in pressure, the dissolving power of nicotine decreases.

The application of the optimal parameters leads to the highest extraction yield. The predicted optimum parameters for the highest extraction yield for the *N. tabacum* cv. Samsun were at 23.41 MPa, at 56.62 °C and 125.1 min extraction time and for the *N. tabacum* cv. Virginia were at 21.03 MPa, at 50.51 °C and 124.1 min extraction time. The SC CO_2_ extraction of *N. tabacum* cv. Samsun and *N. tabacum* cv. Virginia at their predicted optimal parameters for obtaining the highest extraction yield was performed three times. The extraction yield under the predicted optimal conditions yielded for Samsun tobacco leaves 3.07 % ± 0.11 and for Virginia tobacco leaves 2.52 % ± 0.10. The results obtained were compatible with the theoretical model value. The goal of the RSM was to develop the method that can be used for the simulation of the extraction that yields the highest nicotine relative amount. By applying appropriate pressure, temperature, and extraction time, the optimal conditions for obtaining the highest extraction nicotine relative amount in *N. tabacum* cv. Samsun was at the pressure of 17.80 MPa, the temperature of 50.51 °C and 104.5 min extraction time and in *N. tabacum* cv. Virginia, it was at 17.29 MPa, at 50.51 °C and 105.5 min extraction time. The extractions for obtaining the highest nicotine relative amount were completed three times, and it was determined for the Samsun tobacco leaves to be 242.80 ± 0.07 and for Virginia tobacco leaves 32.27 ± 0.13 mg per 100 g of plant material. These values are close to the value of the ideal case. A general inclusive concept revealed that the optimum extraction time was longer compared to previous reports. The phytochemical profile of two *N. tabacum* varieties revealed that the variety Samsum is the best for the recovery of nicotine.

## 4. Materials and Methods

### 4.1. Chemicals

The CO_2_ used for the extraction was 99.97% pure (Messer, Tehnogas AD, Rakovica, Serbia). Nicotine standard (99% purity) was purchased from Sigma-Aldrich Chemie GmbH (Taufkirchen, Germany). All other solvents used were of analytical reagent grade.

### 4.2. Plant Material

*N. tabacum* cv. Samsun was purchased from a local producer in November 2021, Shuakhevi, Adjara, Georgia and *N. tabacum* cv. Virginia from a local producer in Kukujevci, Srem, Serbia. The leaves were air-cured in a well-ventilated barn for three months. Cured tobacco leaves were grounded and sieved for 15 min using a vertical vibratory sieve shaker (Labortechnik GmbH, Ilmenau, Germany). The average particle size was 0.352 mm ± 0.043 for Samsun tobacco leaves and 0.361 mm ± 0.037 for Virginia tobacco leaves. The water content of grounded tobacco leaves was determined according to AOAC Official Method 925.40 and was 2.86 ± 0.11% for Samsun tobacco leaves and 2.37 ± 0.09% for the Virginia tobacco leaves. All measurements were performed in triplicate. The Samsun tobacco leaves and Virginia tobacco leaves powder obtained were used for the SC CO_2_ extractions.

### 4.3. Extraction Procedure

The experiments were completed in a SC CO_2_ system described previously [22]. Here, 50 g of plant material powder was used for the each extraction. The extractions were performed at different extractions conditions determined by CCRD. For all extractions, the CO_2_ mass flow rate was 1.94 kg/h.

### 4.4. Experimental Design

For determining the optimal process parameters of pressure, temperature and time, the CCRD was used [23]. The extraction pressure (X_1_), temperature (X_2_), and time (*X*_3_) were independent variables studied to optimize the extraction process in terms of obtaining a higher total extraction yield and nicotine relative amount. Investigated factors and levels tested are depicted in Table 1.

Experimental data were fitted with the second-order response surface model with the following equation:

(1) $$Y\;=\;\beta_{0}\;+\;\overset{k}{\underset{j\;=\;1}{\sum }}\beta_{j}X_{j}\;+\;\overset{k}{\underset{j\;=\;1}{\sum }}\beta_{jj}X_{j}^{2}\;+\;\sum \underset{i\;<\;j}{\sum }\beta_{ij}X_{i}X_{j}$$

where *Y* is the response variable, *β*_0_ is a constant, *β_j_*, *β_jj_* and *β_ij_* are regression coefficients of the model, and *X_j_* and *X_i_* are the independent variables in coded values. The statistical analysis of experimental data and three-dimensional response surface plots were generated using Minitab LLC^®^, 2021. The test of statistical difference was based on the total error criteria with the confidence level of 95.0%.

### 4.5. GC–MS Analysis

The samples obtained were dissolved in *n*-hexane. The GC–MS analyses were carried out on Agilent 7890B GC fitted with a mass selective detector 5977A (Agilent Technologies, Palo Alto, CA, USA). The capillary column was HP-5MS (5% phenyl-methyl polysiloxane, 30m × 250 μm × 0.25 μm). Helium was the carrier gas at 1 mL·min^−1^. The injection port temperature was 250 °C. The HP-5MS column temperature was programmed at 70 °C isothermal for 2 min and then increased to 200 °C·min^−1^ at the rate of 3 °C·min^−1^ and held isothermal for 20 min. The split ratio was 1:50. The ionization voltage was 70 eV. The ion source temperature was 230 °C. The mass scan range was 60–650 mass units. The injected sample volume was 1 μL. The identification of components was carried out based on computer matching with the NIST 2008 MS library. The percentage composition was calculated from the GC peak areas using the normalization method. The quantitative analysis was completed using calibration curves. Standard compound was dissolved in *n*-hexane, and prepared were six different concentrations of nicotine. The *R*^2^ for the calibration curve was 0.999. All analyses were performed in triplicate.

## 5. Conclusions

The research presents the optimization of SC CO_2_ extraction of dried *N. tabacum* cv. Samsun and *N. tabacum* cv. Virginia leaves. The results of the statistical assays showed that the pressure, temperature, and time have a significant effect on the total extraction yield and pressure and time on nicotine relative amount. The differences in nicotine relative amount indicated that the quantity depends on the variety. The two varieties have different phytochemical compounds, indicating that the abundance of phytochemical compounds depends on the variety. The optimal temperature for the SC CO_2_ extraction for the highest nicotine relative amount is the same and the optimal pressure and time are slightly different. Further investigations can give a better understanding of parameters influencing the total extraction yield and nicotine relative amount in *N. tabacum* varieties.

## Figures and Tables

**Table 1:** The uncoded and coded levels of independent variables used in the RSM.

		Levels				
Independent Variables	Symbol	−1.414	−1	0	+1	+1.414
Pressure [MPa]	*X*_1_	8	10	15	20	22
Temperature [°C]	*X*_2_	36	40	50	60	64
Time [min]	*X_3_*	39.55	60	90	120	140.45

**Table 2:** The CCRD experimental design and results for the *N. tabacum* cv. Samsun and *N. tabacum* cv. Virginia leaves total extraction yield [%] for the response surface analysis.

No.	Pressure [MPa]	Temperature [°C]	Time [min]	Extraction Yield *N. tabacum* cv. Samsun [%]	Extraction Yield *N. tabacum* cv. Virginia [%]
1.	10	40	60	0.82	0.75
2.	20	40	60	1.03	0.92
3.	10	60	60	0.11	0.08
4.	20	60	60	1.45	1.19
5.	10	40	120	1.73	1.67
6.	20	40	120	2.10	1.98
7.	10	60	120	0.51	0.32
8.	20	60	120	2.76	2.23
9.	6.59	50	90	0.38	0.21
10.	23.41	50	90	2.99	2.33
11.	15	33.18	90	1.96	1.88
12.	15	66.82	90	0.92	0.75
13.	15	50	39.55	0.63	0.37
14.	15	50	140.45	2.36	2.26
15.	15	50	90	2.22	1.95
16.	15	50	90	2.17	1.90
17.	15	50	90	2.31	2.11
18.	15	50	90	2.28	2.14
19.	15	50	90	2.07	1.89
20.	15	50	90	2.19	1.96

**Table 3:** The response surface regression coefficients of the polynomial function for the *N. tabacum* cv. Samsun and *N. tabacum* cv. Virginia leaves total extraction yield [%].

Term	Coefficient	Standard Error Coefficient	*T*-Value	*p*-Value
Extraction yield *N. tabacum* cv. Samsun				
Constant	2.2127	0.0980	22.57	0.000
*X*_1_	0.6268	0.0651	9.63	0.000
*X*_2_	−0.1903	0.0651	−2.93	0.015
*X*_3_	0.4832	0.0651	7.43	0.000
*X*_1_·*X*_1_	−0.2236	0.0633	−3.53	0.005
*X*_2_·*X*_2_	−0.3103	0.0633	−4.90	0.001
*X*_3_·*X*_3_	−0.2908	0.0633	−4.59	0.001
*X*_1_·*X*_2_	0.3762	0.0850	4.43	0.001
*X*_1_·*X*_3_	0.1338	0.0850	1.57	0.147
*X*_2_·*X*_3_	−0.0338	0.0850	−0.40	0.700
*R*^2^ = 0.9578				
Extraction yield *N. tabacum* cv. Virginia				
Constant	1.9954	0.0845	23.60	0.000
*X*_1_	0.5174	0.0561	9.22	0.000
*X*_2_	−0.2490	0.0561	−4.44	0.001
*X*_3_	0.4715	0.0561	8.40	0.000
*X*_1_·*X*_1_	−0.2795	0.0546	−5.12	0.000
*X*_2_·*X*_2_	−0.2636	0.0546	−4.83	0.001
*X*_3_·*X*_3_	−0.2636	0.0546	−4.83	0.001
*X*_1_·*X*_2_	0.3175	0.0733	4.33	0.001
*X*_1_·*X*_3_	0.1175	0.0733	1.60	0.140
*X*_2_·*X*_3_	−0.0875	0.0733	−1.19	0.260
*R*^2^ = 0.9628				

**Table 4:** The ANOVA for the response surface square model for the *N. tabacum* cv. Samsun and *N. tabacum* cv. Virginia leaves total extraction yield [%] obtained by SC CO_2_ extraction.

No.	Source	Adjusted Sum of Squares	Adjusted Mean Squares	*F*-Value	*p*-Value	Adjusted Sum of Squares	Adjusted Mean Squares	*F*-Value	*p*-Value
		cv. Samsun				cv. Virginia			
Model	9	13.1254	1.45838	25.24	0.000	11.1270	1.23633	28.77	0.000
Linear	3	9.0484	3.01615	52.19	0.000	7.5375	2.51249	58.46	0.000
*X*_1_	1	5.3647	5.36468	92.83	0.000	3.6553	3.65530	85.06	0.000
*X*_2_	1	0.4946	0.49463	8.56	0.015	0.8467	0.84667	19.70	0.001
*X*_3_	1	3.1891	3.18913	55.18	0.000	3.0355	3.03550	70.63	0.000
Square	3	2.7922	0.93074	16.11	0.000	2.6113	0.87044	20.25	0.000
*X*_1_·*X*_1_	1	0.7208	0.72083	12.47	0.005	1.1261	1.12606	26.20	0.000
*X*_2_·*X*_2_	1	1.3873	1.38732	24.01	0.001	1.0015	1.00152	23.31	0.001
*X*_3_·*X*_3_	1	1.2189	1.21887	21.09	0.001	1.0015	1.00152	23.31	0.001
2-Way Interaction	3	1.2847	0.42825	7.41	0.007	0.9782	0.32605	7.59	0.006
*X*_1_·*X*_2_	1	1.1325	1.13251	19.60	0.001	0.8065	0.80645	18.77	0.001
*X*_1_·*X*_3_	1	0.1431	0.14311	2.48	0.147	0.1105	0.11045	2.57	0.140
*X*_2_·*X*_3_	1	0.0091	0.00911	0.16	0.700	0.0612	0.06125	1.43	0.260
Error	10	0.5779	0.05779			0.4297	0.04297		
Lack-of-Fit	5	0.5414	0.10827	14.82	0.005	0.3723	0.07445	6.48	0.031
Pure Error	5	0.0365	0.00731			0.0575	0.01150		
Total	19	13.7033				11.5567			

**Table 5:** The relative amount (in mg nicotine equivalents per 100 g of plant material) of compounds in SC CO_2_ extracts of *N. tabacum* cv. Samsun leaves.

No.	Compound	Run 1	Run 2	Run 3	Run 4	Run 5	Run 6	Run 7	Run 8	Run 9	Run 10	Run 11	Run 12	Run 13	Run 14	Run 15
1	*trans*-Anethole	0.7	0.0	0.0	0.0	2.0	0.0	0.2	0.0	0.9	0.0	0.4	1.2	0.0	1.6	1.4
2	Nicotine	38.1	62.2	64.2	95.1	119.3	161.0	109.8	185.3	148.1	222.3	102.7	118.6	7.3	217.2	242.1
3	*β*-Damascenone	3.9	0.0	0.0	0.0	8.8	0.0	0.0	0.0	5.2	0.0	5.4	0.0	2.5	7.3	6.7
4	Butylhydroxytoluene	0.1	0.0	0.2	0.0	0.5	0.0	0.5	0.0	0.0	0.0	0.1	0.5	0.0	0.3	0.2
5	3-oxo-*α*-ionol	3.5	0.0	0.0	0.0	7.5	0.0	0.0	0.0	1.7	0.0	9.2	0.0	0.0	0.0	0.0
6	Neophytadiene	10.4	14.7	7.7	14.3	22.3	30.9	17.7	31.7	28.0	36.8	21.8	24.1	17.8	36.5	32.0
7	Hexahydrofarnesol	0.2	0.0	0.0	0.0	0.6	0.0	0.1	0.0	0.3	0.0	0.1	1.5	0.2	1.2	0.9
8	Thunbergol	1.6	3.8	0.1	3.7	4.0	6.5	0.5	9.0	0.0	0.0	0.0	9.1	4.0	8.6	7.8
9	Sclareolide	0.0	0.9	0.0	0.9	0.0	2.1	0.0	2.1	0.0	1.5	1.3	1.8	0.2	1.7	1.5
10	Phytol	0.5	0.0	0.6	0.0	1.5	0.0	1.7	0.0	2.2	0.0	0.7	0.0	1.1	2.5	2.3
11	Agatholic acid	0.0	0.3	0.0	0.0	0.0	1.0	0.0	0.0	0.0	0.9	0.0	0.0	0.0	0.0	0.0
12	(3*β*)-Stigmast-5-en-3-ol	0.0	0.2	0.0	0.2	0.0	0.7	0.0	0.5	0.0	0.3	0.0	0.0	0.0	0.0	0.0

**Table 6:** The relative amount (in mg nicotine equivalents per 100 g of the plant material) of compounds in SC CO_2_ extracts of *N. tabacum* cv. Virginia leaves.

No.	Compound	Run 1	Run 2	Run 3	Run 4	Run 5	Run 6	Run 7	Run 8	Run 9	Run 10	Run 11	Run 12	Run 13	Run 14	Run 15
1	Nicotine	5.2	8.1	8.4	11.7	15.3	20.6	14.2	22.7	19.3	28.9	13.3	14.7	0.8	30.2	32.4
2	Solanone	1.2	1.7	0.2	0.5	1.6	2.1	0.5	0.7	1.4	2.6	1.9	3.3	0.0	2.1	2.0
3	Norsolanadione	0.5	0.7	0.3	0.3	0.8	1.2	0.6	0.5	0.7	1.4	1.7	1.5	0.0	1.3	0.9
4	3-oxo-*α*-ionol	0.6	0.0	0.0	0.0	0.9	0.0	0.0	0.0	0.4	0.0	1.7	0.0	0.0	2.1	1.9
5	Farnesol	2.8	2.6	0.4	0.5	3.7	3.5	0.9	0.7	2.2	6.2	8.3	5.5	0.5	5.2	4.9
6	Neophytadiene	7.5	9.3	5.1	9.1	8.8	10.9	8.6	11.3	19.9	24.4	13.3	14.7	1.9	22.6	20.2
7	5-nonadecene	0.0	0.1	0.0	0.2	0.0	0.3	0.0	0.3	0.0	1.2	1.3	0.9	0.0	1.8	0.8
8	Thunbergol	2.6	5.5	0.3	6.1	3.7	6.7	0.7	8.4	0.0	1.3	0.0	12.9	2.6	13.5	11.1
9	Methyl linoleate	0.0	0.8	0.0	0.8	0.0	1.3	0.0	1.2	0.0	3.8	4.3	3.1	0.0	4.2	3.4
10	Sclareolide	0.0	0.3	0.0	0.1	0.0	0.5	0.0	0.2	0.0	1.0	1.3	0.6	0.0	2.0	1.1
11	1-docosene	0.0	2.2	0.0	4.4	0.0	2.8	0.0	7.5	0.0	5.4	5.0	5.6	0.9	5.1	4.3
12	Geranyl geraniol	0.1	0.2	0.0	0.0	0.3	0.5	0.0	0.0	0.0	0.6	0.1	0.5	0.0	0.7	0.4
13	4,8,13-duvatriene-1,3-diol	0.0	0.0	1.1	2.4	0.0	0.0	2.3	3.7	3.9	3.9	0.0	0.0	0.0	0.0	0.0
14	8,13-epoxy-14-labden-12-ol	95.7	98.8	34.8	42.8	102.8	110.5	54.8	59.1	105.1	180.1	228.3	168.6	12.8	271.3	262.4
15	(*E*)-stigmasta-5,22-dien-3*β*-ol	0.0	0.2	0.0	0.3	0.0	0.4	0.0	0.5	0.0	1.6	0.0	0.3	0.0	0.0	0.0
16	Triacontyl acetate	0.5	0.1	2.0	1.1	1.1	0.2	3.1	1.8	0.0	2.2	0.0	2.0	0.0	2.2	1.8
17	Solanesol	6.1	8.3	0.5	9.2	7.3	9.7	8.2	11.3	18.2	24.4	5.1	15.0	1.6	16.2	6.3

**Table 7:** The response surface regression coefficients of the polynomial function for the *N. tabacum* cv. Samsun and *N. tabacum* cv. Virginia leaves’ nicotine relative amount (mg nicotine equivalents per 100 g of plant material).

Term	Coefficient	Standard Error Coefficient	*T*-Value	*p*-Value
Nicotine relative amount *N. tabacum* cv. Samsun				
Constant	234.41	7.83	29.94	0.000
*X*_1_	21.26	5.19	4.09	0.002
*X*_2_	6.71	5.19	1.29	0.225
*X*_3_	47.05	5.19	9.06	0.000
*X*_1_·*X*_1_	−22.39	5.06	−4.43	0.001
*X*_2_·*X*_2_	−48.75	5.06	−9.64	0.000
*X*_3_·*X*_3_	−50.01	5.06	−9.89	0.000
*X*_1_·*X*_2_	4.24	6.79	0.62	0.546
*X*_1_·*X*_3_	6.94	6.79	1.02	0.331
*X*_2_·*X*_3_	−6.64	6.79	−0.98	0.351
*R*^2^ = 0.9659				
Nicotine relative amount *N. tabacum* cv. Virginia				
Constant	31.46	1.28	24.58	0.000
*X*_1_	2.647	0.849	3.12	0.011
*X*_2_	0.744	0.849	0.88	0.402
*X*_3_	6.506	0.849	7.66	0.000
*X*_1_·*X*_1_	−3.354	0.827	−4.06	0.002
*X*_2_·*X*_2_	−6.925	0.827	−8.38	0.000
*X*_3_·*X*_3_	−6.394	0.827	−7.74	0.000
*X*_1_·*X*_2_	0.45	1.11	0.41	0.694
*X*_1_·*X*_3_	0.95	1.11	0.86	0.412
*X*_2_·*X*_3_	−0.73	1.11	−0.65	0.528
*R*^2^ = 0.9514				

**Table 8:** The ANOVA for the response surface square model for the *N. tabacum* cv. Samsun and *N. tabacum* cv. Virginia leaves’ nicotine relative amount.

No.	Source	Adjusted Sum of Squares	Adjusted Mean Squares	*F*-Value	*p*-Value	Adjusted Sum of Squares	Adjusted Mean Squares	*F*-Value	*p*-Value
		cv. Samsun				cv. Virginia			
Model	5	104,509	11,612.1	31.51	0.000	1927.23	214.136	21.75	0.000
Linear	3	37,020	12,340.1	33.49	0.000	681.19	227.065	23.06	0.000
*X*_1_	1	6170	6170.4	16.75	0.002	95.66	95.665	9.72	0.011
*X*_2_	1	615	614.9	1.67	0.225	7.55	7.550	0.77	0.402
*X*_3_	1	30,235	30,235.1	82.06	0.000	577.98	577.980	58.70	0.000
Square	3	66,607	22,202.4	60.26	0.000	1232.99	410.995	41.74	0.000
*X*_1_·*X*_1_	1	7227	7227.3	19.61	0.001	162.11	162.111	16.47	0.002
*X*_2_·*X*_2_	1	34,252	34,251.7	92.96	0.000	691.07	691.068	70.19	0.000
*X*_3_·*X*_3_	1	36,038	36,038.0	97.80	0.000	589.27	589.272	59.85	0.000
2-Way Interaction	3	881	293.7	0.80	0.523	13.05	4.348	0.44	0.728
*X*_1_·*X*_2_	1	144	143.7	0.39	0.546	1.62	1.620	0.16	0.694
*X*_1_·*X*_3_	1	385	385.0	1.04	0.331	7.22	7.220	0.73	0.412
*X*_2_·*X*_3_	1	352	352.5	0.96	0.351	4.21	4.205	0.43	0.528
Error	10	3685	368.5			98.46	9.846		
Lack-of-Fit	5	3555	711.1	27.47	0.001	96.18	19.237	42.31	0.000
Pure Error	5	129	25.9			2.27	0.455		
Total	19	108,193				2025.68			

## Data Availability

Not applicable.

## Sample Availability

Samples of the extracts are available from the author.
